# Metal-dependent electrochemical discrimination of DNA quadruplex sequences

**DOI:** 10.1007/s00775-021-01881-9

**Published:** 2021-08-04

**Authors:** Daniela Escher, M. Nur Hossain, Heinz-Bernhard Kraatz, Jens Müller

**Affiliations:** 1 Institut für Anorganische und Analytische Chemie, Westfälische Wilhelms-Universität Münster, Corrensstr. 30, 48149 Münster, Germany; 2 Department of Physical and Environmental Sciences, University of Toronto Scarborough, 1265 Military Trail, Toronto, M1C 1A4 Canada

**Keywords:** Quadruplex DNA, DNA topology, Metal-binding, Electrochemistry

## Abstract

Films of four different DNA quadruplex-forming (G4) sequences (*c-KIT*, *c-MYC*, HTelo, and *BCL2*) on gold surfaces were investigated by electrochemical impedance spectroscopy (EIS) to evaluate whether they evoke unique electrochemical responses that can be used for their identification. This could render EIS an alternative means for the determination of G4 sequences of unknown structure. Towards, this end, cation-dependent topology changes in the presence of either K^+^, K^+^ in combination with Li^+^, or Pb^2+^ in the presence of Li^+^ were first evaluated by circular dichroism (CD) spectroscopy, and electrochemical studies were performed subsequently. As a result, G4-sequence specific charge transfer resistance (*R*_CT_) patterns were in fact observed for each G4 sequence, allowing their discrimination by EIS.

**Graphic abstract:**

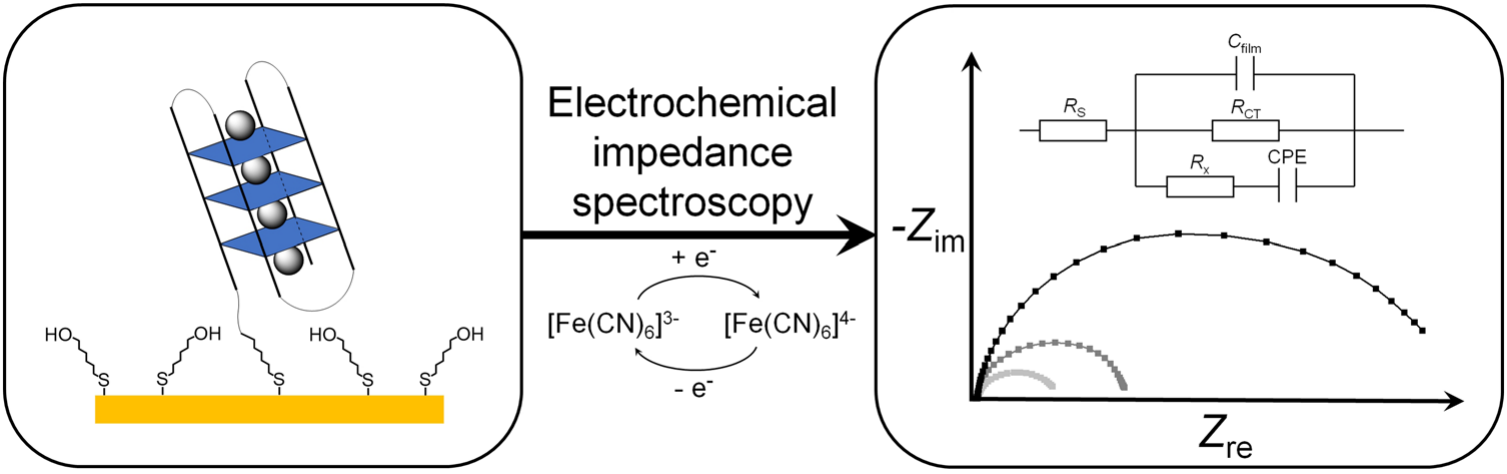

**Supplementary Information:**

The online version contains supplementary material available at 10.1007/s00775-021-01881-9.

## Introduction

**Fig. 1:**
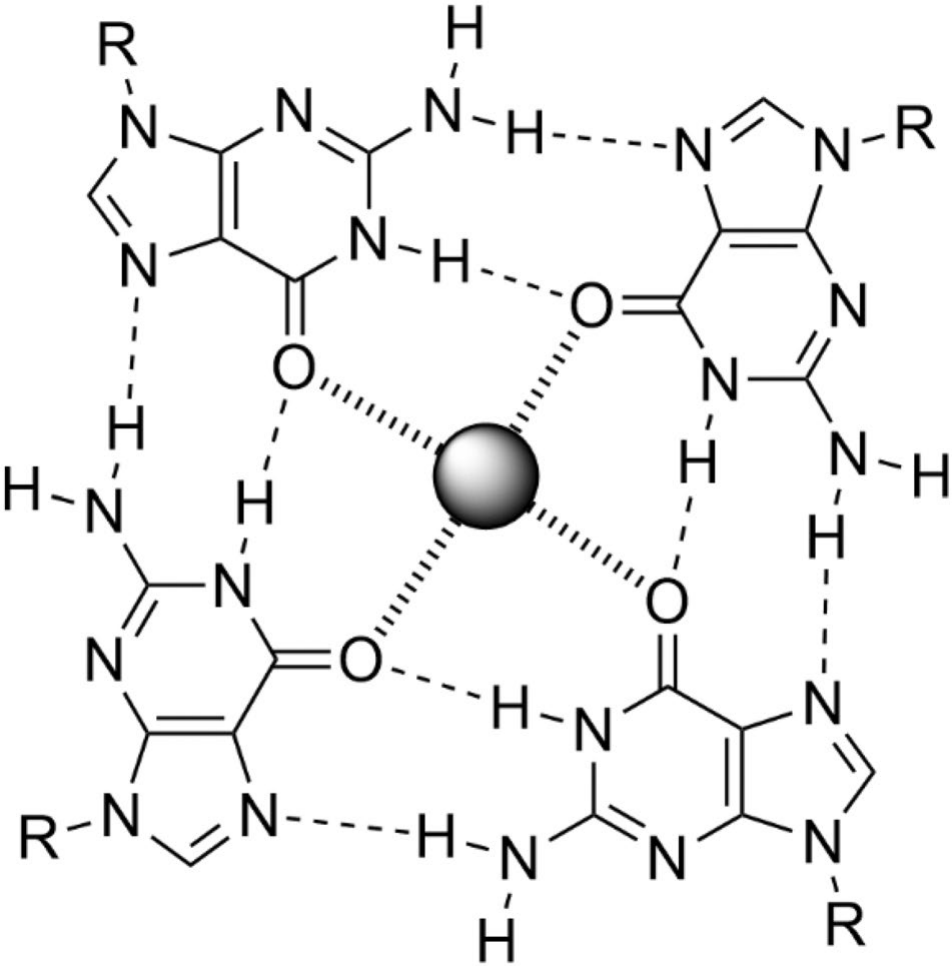
Schematic representation of a tetrad formed by four guanine residues. A black sphere represents the central cation. R = DNA backbone

Guanine-rich oligonucleotide sequences can adopt tetra-stranded structures referred to as quadruplex (G4) DNA [1]. The formation of G4 DNA is based on the self-assembly of four guanine (G) nucleobases in the form of tetrads, with a centrally positioned (mostly monovalent) cation coordinating the guanine keto groups (Fig. 1) [2]. Within G4 DNA, individual tetrads are located adjacent to each other, allowing the nucleobases to interact via π stacking. G4 DNA has been identified as a potential target in a novel approach to cancer therapy [3]. Moreover, the triggered formation of G4 DNA has been successfully applied to develop sensors for the respective trigger molecules [4]. Finally, it is an important player in DNA nanotechnology [5].

Structural studies have shown that G4 DNA can exist in a variety of topologies [6]. Circular dichroism (CD) spectroscopy represents a prominent tool to distinguish between these topologies [7]. The quadruplex topology is affected by the presence and the identity of its central cations. Among the alkali and alkaline earth metal ions, K^+^ ions exhibit the strongest G4 stabilization, whereas Li^+^ and Cs^+^ ions are only weakly stabilizing [8–15]. In addition, further cations including NH_4_^+^ [16], Tb^3+^ [17] or transition metal ions such as Co^2+^, Zn^2+^ and other divalent ions [12, 18] have been investigated. Amongst these, Pb^2+^ ions have attracted particular attention. Several studies have revealed a high affinity of Pb^2+^ towards G4 DNA [19–22].

The aim of this study is to evaluate whether electrochemical impedance spectroscopy (EIS) may act as a means to determine the identity of a given G4 DNA sequence. Towards this end, the charge transfer resistance (*R*_CT_) of a film of surface-deposited G4 DNA was measured under different experimental conditions and correlated with the corresponding CD spectroscopic results.

## Materials and methods

G4 DNA oligonucleotides were synthesized using automated solid-phase synthesis and purified as reported previously [23]. All phosphoramidites were purchased from Glen Research. The identity of the desired G4 sequences was confirmed by MALDI-TOF mass spectrometry.

CD-spectroscopic studies were performed on a *J-815* CD spectrometer (Jasco) using a quartz cuvette with 1 cm path length at 5 °C. The G4 solutions contained 1 μM DNA, 5 mM 3-(*N*-morpholino)propanesulfonic acid (MOPS), adjusted with either KOH or LiOH to pH 6.8, and 60 mM KCl or LiClO_4_, respectively. For measurements in the presence of Pb^2+^, Pb(NO_3_)_2_ was used.

Electrochemical impedance spectroscopy studies and cleaning of the electrodes were performed as reported previously [24–27]. The measurement solution comprised 2 mM K_4_[Fe(CN)_6_] and 2 mM K_3_[Fe(CN)_6_], 5 mM MOPS (pH 6.8), and 60 mM KCl or LiClO_4_. Freshly cleaned gold electrodes were incubated in a 25 μM aqueous solution of G4 DNA in either K^+^- or Li^+^-containing buffer (5 mM MOPS (pH 6.8), 60 mM KCl or LiClO_4_). After incubation for 3 d at 5 °C, electrodes were carefully washed with the respective buffer solution and subsequently incubated in MCH (1 mM in K^+^- or Li^+^-containing buffer) for 1 h at room temperature.

Nyquist plots of the obtained EIS data were fitted by an equivalent circuit (R(CR(R(CPE))) with R = resistor, C = capacitor and CPE = constant phase element) which had been used previously for similar studies (Scheme S3-1, Supp. Inf.) [24–26]. Moreover, preliminary fits using other equivalent circuits (e.g. a Randles circuit without the Warburg element (Scheme S3-2a, Supp. Inf.) or an equivalent circuit in which the capacitor was replaced by a CPE (Scheme S3-2b, Supp. Inf.) have shown that the equivalent circuit considered in this work gives the best fit of experimental and calculated data (Tables S3-3–S3-5, Supp. Inf.). This is particularly true for the fit of the high-frequency region, as can clearly be discerned from the respective Bode plots (Fig. S3-2, Supp. Inf.). Although the equivalent circuit R(CR(R(CPE))) seems to be overfitting the data in a few cases (Tables S3-3 and S3-4, Supp. Inf.), this circuit was used since it is the only one with a good fit of the low- *and* high-frequency regions (Fig. S3-2, Supp. Inf.). Moreover, using the same model throughout allows a better comparison of the parameters.

## Results and discussion

### Choice of nucleic acid sequences

**Fig. 2:**
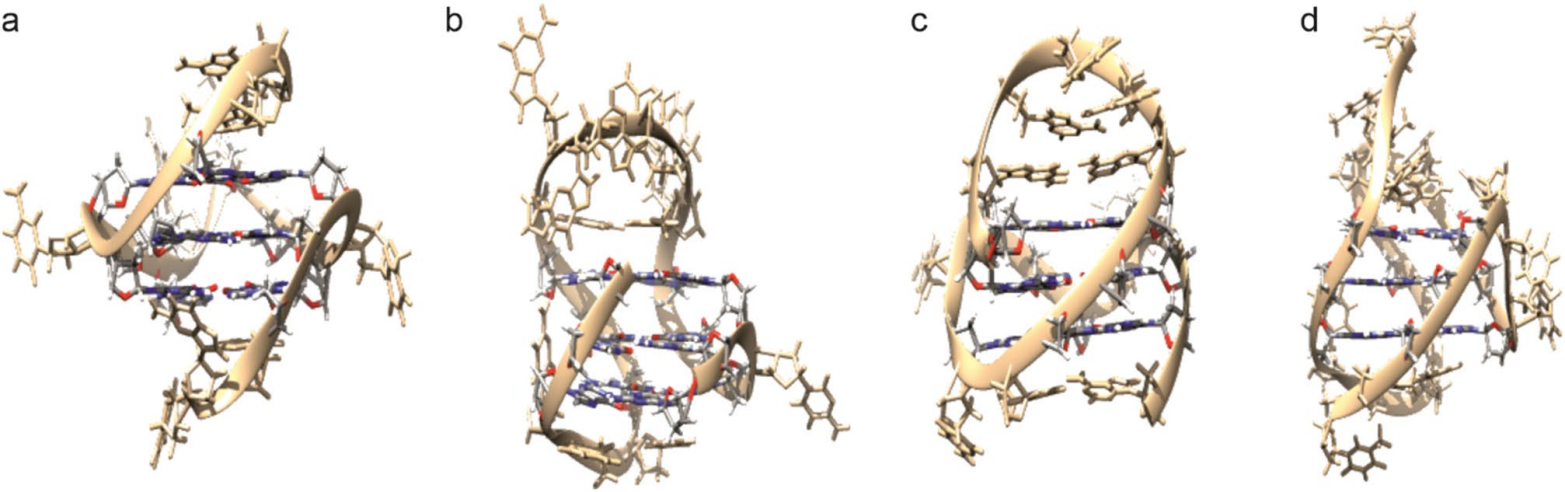
Experimental structures of the G4 sequences used in this study as determined in K^+^-containing solutions. **a** *c-MYC* [31]; **b** *BCL2* [32]; **c** *c-KIT* [28]; **d** HTelo [35]. This figure was created using UCSF Chimera [36]

A total of four G4 DNA sequences were studied by electrochemical impedance spectroscopy. In particular, G-rich sequences from the promoter regions of the *c-KIT* [28, 29], *c-MYC* [30, 31] and *BCL2* [32] genes as well as the human telomeric DNA sequence HTelo [33] were selected (Table 1), as the structures of these G4 DNA sequences are known from single-crystal X-ray diffraction analysis or from solution NMR studies. Moreover, they represent relevant therapeutic targets [34]. While parallel-stranded G4 topologies exist in *c-KIT*, *c-MYC* and HTelo in the presence of K^+^ [28–31, 33], a [3 + 1] strand orientation is known to be adopted by *BCL2* [32]. Their respective experimental structures are depicted in Fig. 2.

**Table 1:** G4 DNA sequences used in this study. G nucleosides involved in the formation of G4 tetrads are highlighted in bold

G4	DNA sequence
*c-KIT*	R-5'-d(TTT A**GG G**A**G GG**C **G**CT **GGG** AGG AG**G G**)
*c-MYC*	R-5'-d(TTT TGA **GGG** T**GG G**TA **GGG** T**GG G**TA A)
HTelo	R-5'-d(TTT A**GG G**TT A**GG G**TT A**GG G**TT A**GG G**)
*BCL2*	R-5'-d(TTT **GGG** CGC **GGG** AGG AAT T**GG G**C**G GG**)

R = disulfide moiety (–(CH_2_)_6_S–S(CH_2_)_6_OH)

### CD-spectroscopic characterization

Prior to the electrochemical characterization of the surface-deposited G4 DNA films, their folding in solution was evaluated by CD spectroscopy. This allows a direct comparison of the charge transfer resistance of the DNA films with more common descriptors such as CD spectra. Due to the cation dependence of the G4 structures, it was decided to investigate the quadruplexes in the presence of different metal cations. In this context, it is important to consider that the redox probe used in the electrochemical studies consists of an equimolar mixture of 2 mM solutions of K_4_[Fe(CN)_6_] and K_3_[Fe(CN)_6_]. Hence, the presence of the excellent G4 binder K^+^ in significant amounts (14 mM) was inevitable. For that reason, a variety of experimental conditions were chosen for the CD spectroscopic and the EIS studies. These are a) the presence of K^+^ ions alone, b) the presence of the poor G4 binder Li^+^ alone, c) a combination of K^+^ and Li^+^ ions, and d) increasing amounts of the strong G4 binder Pb^2+^.

For the CD-spectroscopic characterization, G4 DNA oligonucleotides (*c* = 1 μM) were initially studied in the presence of either K^+^ (60 mM) or Li^+^ (60 mM), respectively, at pH 6.8 (5 mM MOPS buffer). Additional measurements were performed using a mixed solution of 14 mM K^+^ and 60 mM Li^+^. Furthermore, Pb^2+^ titrations were carried out with Li^+^-containing G4 DNA in the absence of K^+^ to identify the concentration required to induce a cation exchange within the G4 quadruplex. Their respective stabilities in the presence of 14 mM K^+^ alone were examined as well. As anticipated, different binding behaviors were observed depending on the G4 sequence.

**Fig. 3:**
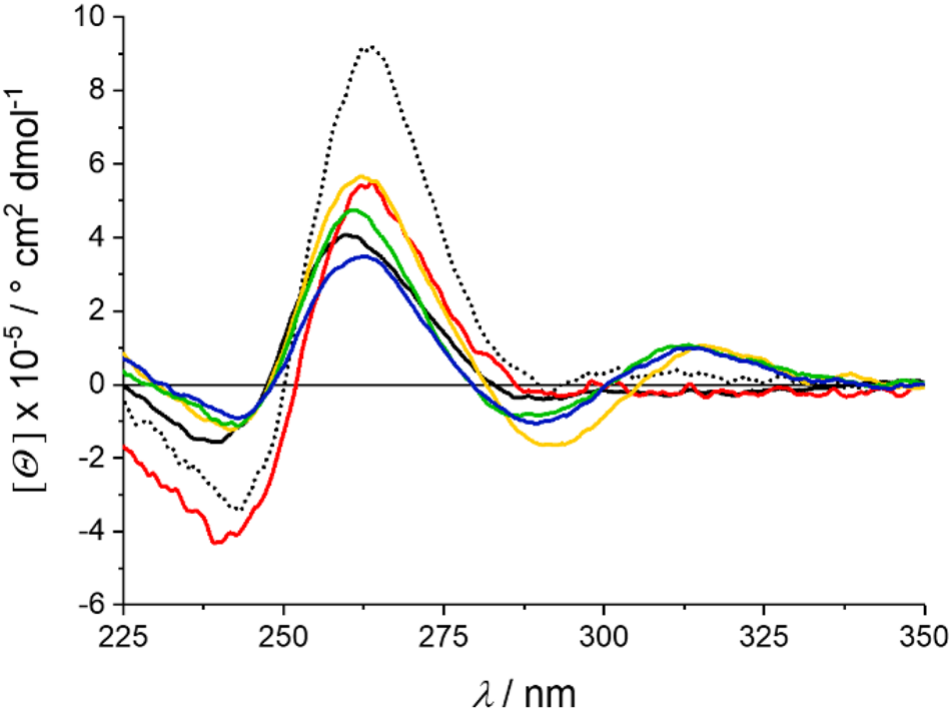
CD spectra of *c-KIT* in the presence of 60 mM K^+^ (dotted black line), 60 mM Li^+^ (solid black line), 14 mM K^+^ and 60 mM Li^+^ (red), or 150 μM Pb^2+^ and 60 mM Li^+^ (yellow), respectively. 14 mM K^+^ was added to the latter and the solution was measured immediately (green) and again after 4 d (blue)

The CD spectra of *c-KIT* are depicted in Fig. 3. The wavelengths of the minima and maxima are almost the same for all measurements in the absence of Pb^2+^, but the intensities vary. This suggests an altering stability but no change of the overall G4 topology. As expected, *c-KIT* DNA is most stable in the presence of K^+^ ions and is destabilized by Li^+^ ions [13]. If both cations are available (K^+^:Li^+^  = 14 mM:60 mM), the G4 structure is stabilized again. However, due to the equilibrium of K^+^ and Li^+^ ions, the quadruplex DNA does not reach the same stability as found for the G4 DNA in the presence of K^+^ only. Upon the addition of Pb^2+^ to the Li^+^-containing G4 DNA, an additional maximum appears at around 315 nm, indicating a cation exchange [19, 22, 25]. Based on this new maximum, it can be concluded that 50 μM Pb^2+^ are required to induce the changes in the case of *c-KIT*. In the presence of 150 μM Pb^2+^, a stable G4 DNA structure is formed (Fig. S2-1, Supp. Inf.), which is again slightly destabilized upon the addition of 14 mM K^+^. Nevertheless, even after four days the characteristic CD signature of G4 DNA with Pb^2+^ at ca. 315 nm remains clearly visible.

**Fig. 4:**
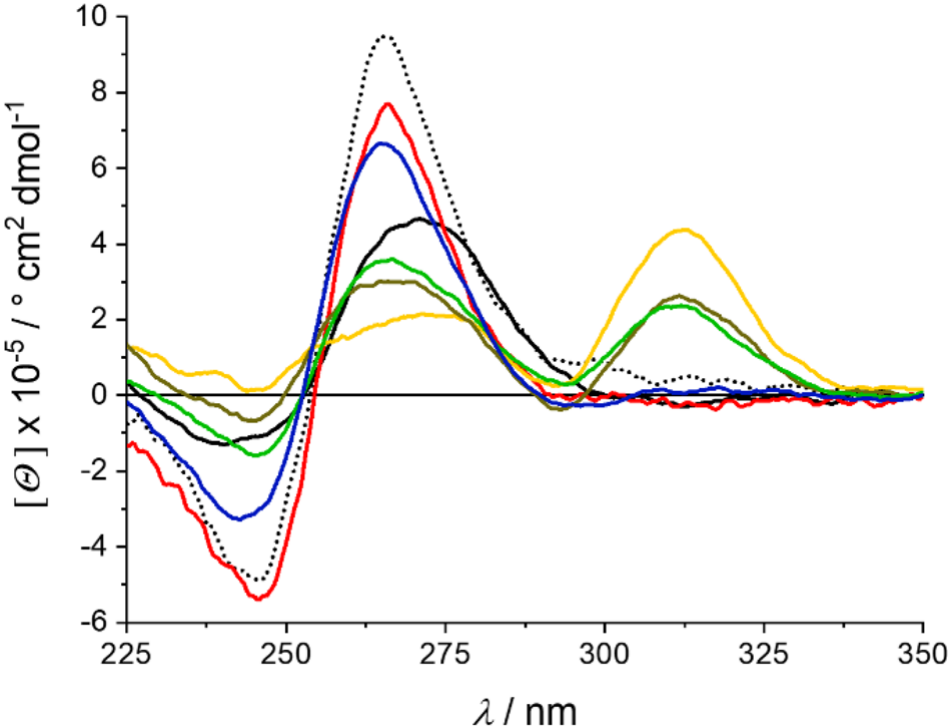
CD spectra of *c-MYC* in the presence of 60 mM K^+^ (dotted black line), 60 mM Li^+^ (solid black line), or 14 mM K^+^ and 60 mM Li^+^ (red). The *c-MYC* G4 solution was measured immediately after the addition of 20 μM Pb^2+^ with 60 mM Li^+^ present (yellow) and again 8 h later (green–brown). Once no further changes were observed, 14 mM K^+^ was added and additional spectra were recorded immediately (green) and after 13 h (blue)

In contrast to *c-KIT*, the CD spectrum of *c-MYC* in the presence of Li^+^ and K^+^ ions more closely resembles that of *c-MYC* with only K^+^ ions (Fig. 4). This indicates higher stability of the K^+^-containing *c-MYC* quadruplex compared to the respective *c-KIT* quadruplex in the presence of a mixture of K^+^ and Li^+^. Interestingly, the maximum at 310 nm, which appears to be characteristic for the interaction between G4 DNA and Pb^2+^, is strongly pronounced immediately after the addition of Pb^2+^ but decreases over time until reaching a constant level after four hours (Fig. S2-2a, Supp. Inf.). This observation is in line with a structural transition via a kinetically favored G4 intermediate towards the thermodynamically favored product. Based on the decline of the maximum at 310 nm, it is likely that different G4 structures resulting from the presence of K^+^, Li^+^ and Pb^2+^ exist at the same time in equilibrium while the formation of the Pb^2+^-containing G4 topology is favored in initially. Such a concentration-dependent coexistence of different G4 DNA topologies with different cations as well as the formation of intermediates with mixed cation occupation has been shown before [37]. Initially, the subsequent addition of 14 mM K^+^ has a minor affect only, but already 15 min later a destabilization of the Pb^2+^-containing G4 is observed (Fig. S2-2b, Supp. Inf.). After 13 h, all Pb^2+^ ions appear to have been replaced by K^+^ and Li^+^ ions, as indicated by the lack of a CD signal at 310 nm and the similarity with the spectrum obtained in the presence of 14 mM K^+^ and 60 mM Li^+^.

**Fig. 5:**
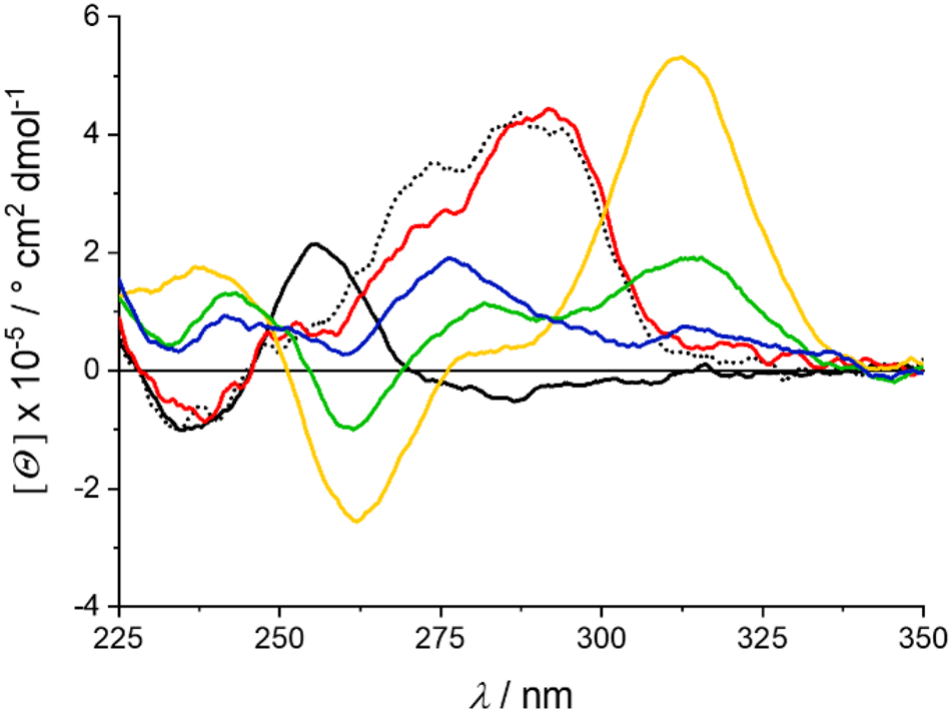
CD spectra of HTelo in the presence of 60 mM K^+^ (dotted black line), 60 mM Li^+^ (solid black line), or 14 mM K^+^ and 60 mM Li^+^ (red). Additional spectra were measured after the subsequent addition of 30 μM Pb^2+^ in the absence (yellow) or presence of 14 mM K^+^, either immediately after the addition of K^+^ (green) of 15 h later (blue)

HTelo G4 DNA undergoes significant topological changes depending on the available cations (Fig. 5). The G4 topology adopted in the presence of Li^+^ is obviously different from the one in the presence of either only K^+^ or K^+^ *and* Li^+^. In contrast to the other three G4 DNA sequences under investigation, a complete change of conformation was observed for HTelo after the addition of 20 μM Pb^2+^ in the presence of Li^+^ and K^+^. Higher concentrations of Pb^2+^ do not affect the G4 structure further (Fig. S2-3, Supp. Inf.). The subsequent addition of 14 mM K^+^ destabilizes that G4 topology immediately, and the changes continue until a stable G4 conformation is reached after 15 h. The final CD spectrum neither fits that of HTelo in the presence of K^+^ or Li^+^ alone nor that obtained in the presence of more than one cation. It can therefore be concluded that in the concurrent presence of Pb^2+^, K^+^ and Li^+^, HTelo adopts a conformation different from those seen before. Based on what has been discussed before, it can be assumed that Li^+^ plays only a minor role here.

**Fig. 6:**
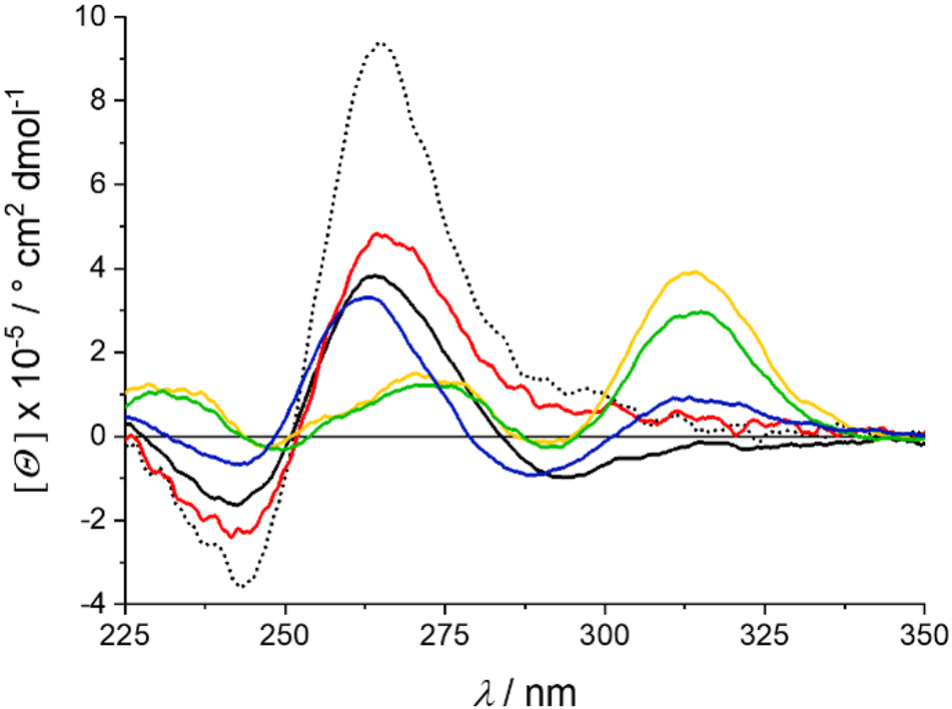
CD spectra of *BCL2* in the presence of 60 mM K^+^ (dotted black line), 60 mM Li^+^ (solid black line), 14 mM K^+^ and 60 mM Li^+^ (red), or 30 μM Pb^2+^ and 60 mM Li^+^. Changes upon the addition of 14 mM K^+^ to the Pb^2+^-containing solution were studied immediately after the addition (green) and after 4 d (blue)

The CD spectra of *BCL2* (Fig. 6) in the absence of Pb^2+^ are similar to those of *c-KIT*. Again, the G4 conformation is affected by the presence of both K^+^ and Li^+^. Upon the addition of Pb^2+^, a time-dependent change of the maximum at 315 nm similar to that seen for *c-MYC* is observed. Furthermore, the changes start to occur after the addition of 10 μM Pb^2+^ for both quadruplexes. However, in contrast to the latter, the maximum is still present after 4 days, but significantly smaller than before.

### Electrochemical impedance spectroscopy

To investigate the differences in *R*_CT_ through the quadruplex film, a solution containing the anionic redox couple [Fe(CN)_6_]^3–/4–^ was used. Earlier studies by scanning electrochemical microscopy and EIS of DNA films on Au surfaces using the [Fe(CN)_6_]^3–/4–^ redox couple demonstrated that metal interactions with DNA give rise to reproducible changes in the diffusion of the redox probe in and out of the thin film [38, 39]. The impedance of DNA films is highly sensitive to such changes and thus has been used extensively to study DNA films including their interaction with metal ions [26].

**Fig. 7:**
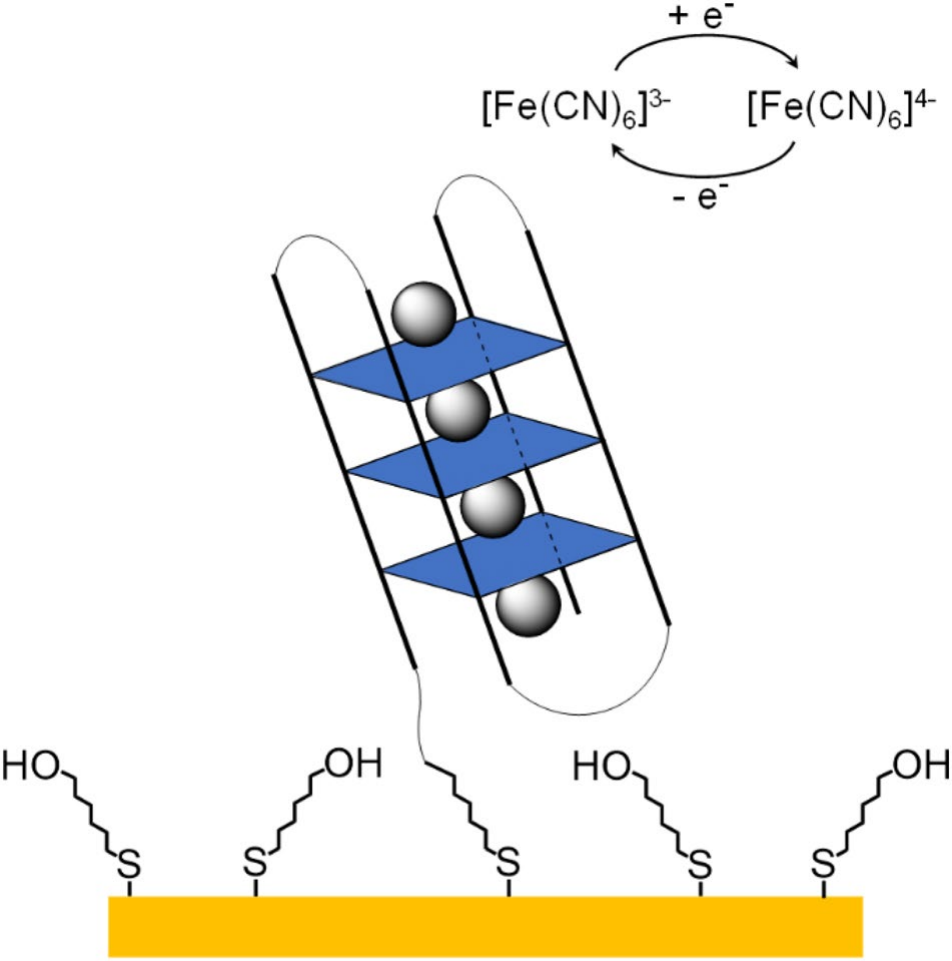
Schematic representation of a backfilled G4 DNA film on a Au electrode surface. Electrochemical experiments were performed using a [Fe(CN)_6_]^3–/4–^ redox probe

Thin films of quadruplex DNA on Au electrodes were prepared by immersing the Au electrodes into disulfide-modified DNA solution. The 5ʹ-disulfide group ensures chemisorption of the oligonucleotides onto the Au surface [24, 40–46]. Next, 6-mercaptohexanol (MCH) was used to backfill the DNA film to achieve a more uniform orientation of the oligonucleotides and to reduce potential film defects and pinholes [27, 43, 45]. The result is a heterogeneous thin film, and a schematic representation of the modified Au surface is depicted in Fig. 7. The fact that the DNA is indeed attached to the surface can be deduced from the observation that a DNA-free MCH-covered Au electrode shows an electrochemical response distinct from that of the DNA-covered and MCH-backfilled surface (Fig. S3-6, Supp. Inf.).

**Fig. 8:**
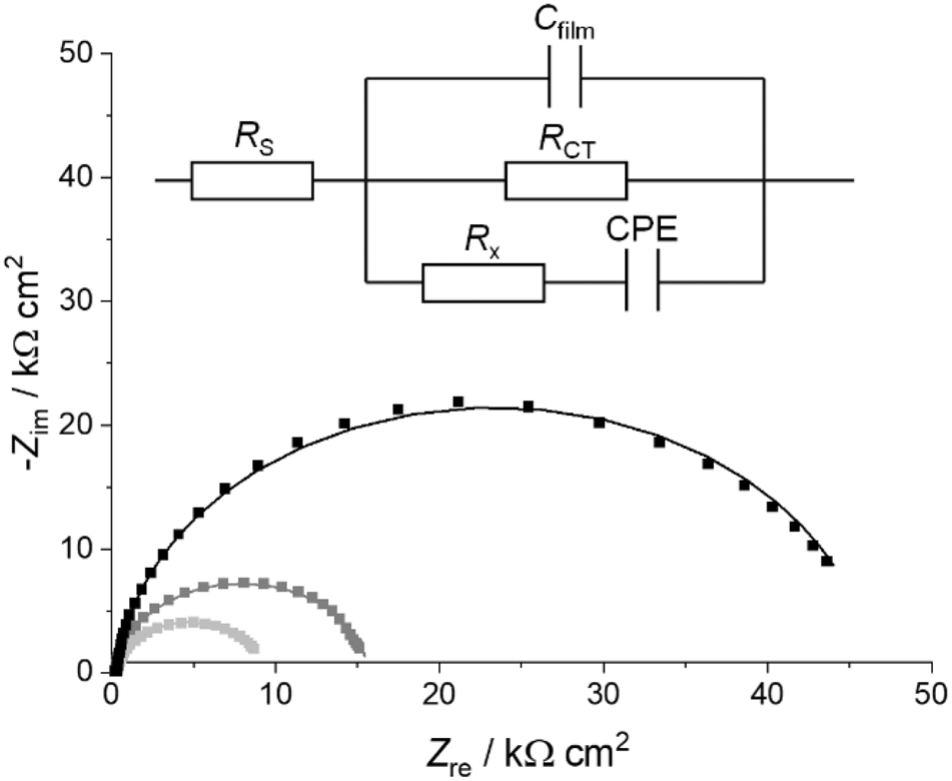
Representative Nyquist plots of a gold surface modified with c-*KIT* G4 DNA in the presence of K^+^-containing buffer solution (gray), of Li^+^-containing buffer solution before (light gray) and after incubation (black) of the latter in Pb^2+^-containing solution. Experimental data (■) are fitted with calculated impedance curves based on the circuit model shown in the inset (solid lines). For details, please see Table S3-1 (Supp. Inf.)

**Fig. 9:**
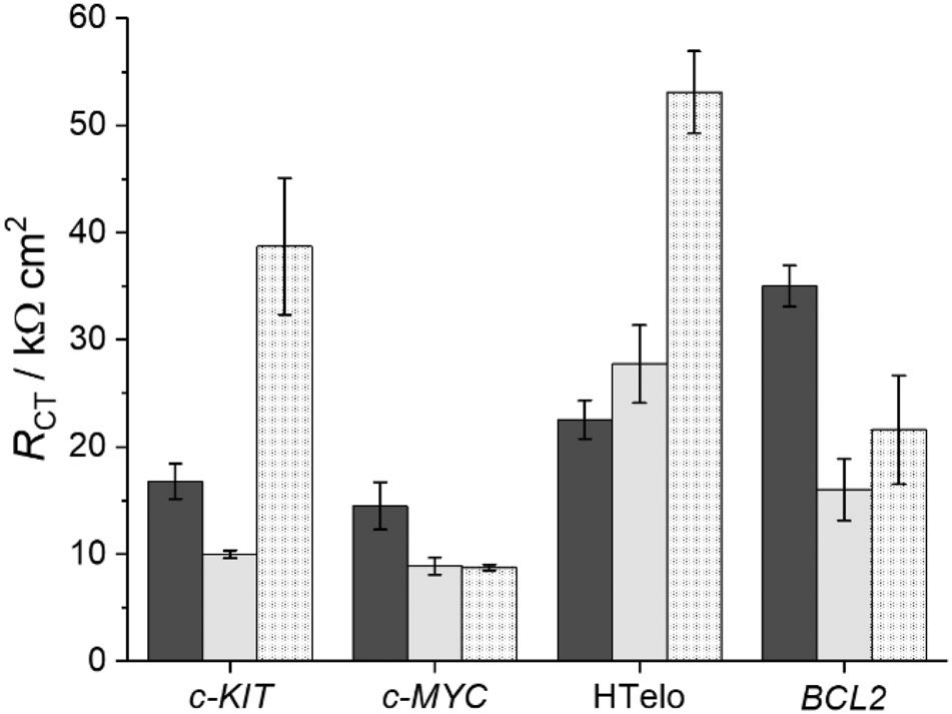
*R*_CT_ values for G4 films on Au electrodes in the presence of K^+^-containing buffer solution (black), Li^+^-containing buffer solution (gray) or Li^+^-containing buffer solution in the presence of Pb^2+^ (dotted). The Li^+^-containing buffer inevitably comprises 14 mM K^+^ due to the presence of K_4_[Fe(CN)_6_] and K_3_[Fe(CN)_6_] in the measurement solution. Data represent an average of three individual measurements

The trends observed for the different G4 DNA oligonucleotides in the EIS studies resemble those of the CD spectroscopic experiments. Representative Nyquist plots (− *Z*_im_ vs. *Z*_re_) together with fits to an equivalent circuit for thin films of *c-KIT* are shown in Fig. 8 and show a significant change in the impedance as a result of K^+^, Li^+^, and Pb^2+^ interactions with the G4 film. Fitting of the electrochemical impedance using a modified Randles’ equivalent circuit allows extracting the charge transfer resistance *R*_CT_ as well as the film capacitance *C*_film_. The equivalent circuit contains a constant phase element acting as a non-ideal capacitor. This is commonly used to account for the heterogeneity of the film on polycrystalline electrodes [47]. *R*_x_ is attributed to residual pinholes and defects in the film structure. All Nyquist plots are given in the Supporting Information (Fig. S3-3–S3-5), the electrochemical impedance parameters are summarized in Table S3-1 (Supp. Inf.). Solution resistances *R*_S_ are not included as they are negligible (*R*_S_ = 10–60 Ω cm^2^). Changes in the *R*_CT_ are responsible for the overall changes in the film impedance and in the following, the changes in *R*_CT_ induced by the presence of different cations to the four G4 DNA sequences under investigation will be discussed (Fig. 9).

The charge transfer resistance of DNA films composed of *c-KIT*, *c-MYC,* and *BCL2* is reduced in the presence of both Li^+^ and K^+^ compared to K^+^ alone. Only the DNA films containing HTelo show almost identical *R*_CT_ values in the absence and presence of Li^+^, taking into account the respective standard deviations. This matches the findings of the CD-spectroscopic studies. While the CD spectra of *c-KIT*, *c-MYC,* and *BCL2* in the presence of Li^+^ and K^+^ differ from those recorded for only K^+^, they are comparable in the case of HTelo. This also confirms that the influence of the different anions (chloride *vs.* perchlorate) is negligible. Interestingly, *R*_CT_ values for *c-KIT* and *c-MYC* are indistinguishable in the presence of buffers containing either K^+^ or K^+^ and Li^+^. *BCL2* on the other hand shows a significantly higher charge transfer resistance *R*_CT_ in both buffers.

After incubation with Pb^2+^, an increase in *R*_CT_ is observed for *c-KIT*, HTelo and *BCL2*, albeit rather small in the latter case. This increase in *R*_CT_ is in good agreement with prior studies [25, 26, 48]. However, no change is observed for *c-MYC*. This finding is not unexpected, because the G4 topology in the presence of Pb^2+^ is relatively unstable, leading to an almost full recovery of the G4 topology present in Li^+^-containing buffer solution with K^+^ (Fig. 4). While it took 13 h to reach this conformation in solution (as derived from the CD spectra), a similar effect was observed electrochemically after a shorter period of time. It can be speculated that the immobilization of *c-MYC* DNA on Au surface affects either the binding affinity or the binding kinetics. This could also be an explanation for the unexpectedly low increase in *R*_CT_ for *BCL2*. If the respective metal-binding capabilities are indeed the reason for the differences in the electrochemical response, then it can be assumed that either the films of *c-KIT* and HTelo quadruplexes release their Pb^2+^ cations more slowly or that the G4 conformations adopted in the presence of Pb^2+^ are more stable for films of *c-KIT* and HTelo compared to *c-MYC* and *BCL2*. Overall, these sequence-dependent differences in *R*_CT_ enable electrochemical discrimination of *c-KIT*, *c-MYC*, HTelo and *BCL2* with a set of three measurements (K^+^, Li^+^, and Pb^2+^).

## Conclusion

We have shown that cation-related conformational changes of the G4-forming oligonucleotide sequences *c-KIT*, *c-MYC*, HTelo, and *BCL2* cannot only be followed by CD spectroscopy, but also by electrochemical impedance spectroscopy using films of G4 DNA attached to a gold surface. Each G4 sequence shows an individual characteristic binding behavior towards K^+^, Li^+^ or Pb^2+^, resulting in sequence-specific changes of the charge transfer resistance *R*_CT_. In addition, significant differences in *R*_CT_ in the presence of Pb^2+^ were observed. While *c-MYC* has a high affinity towards K^+^, leading to a fast cation exchange of Pb^2+^ by K^+^ and hence no change in *R*_CT_, CD-spectroscopic and EIS studies of *c-KIT*, HTelo and *BCL2* confirm that their G4 conformations are reasonably affected by the presence of Pb^2+^. As less Pb^2+^ was needed to induce conformational changes in HTelo compared to *c-KIT*, the former sequence may be more sensitive towards Pb^2+^, even though the percentaged change of *R*_CT_ upon the addition of Pb^2+^ was higher for *c-KIT*. As summarized in Table 2, a combination of three measurements of *R*_CT_ in the presence of different combinations of cations represents a helpful tool for identifying the G4 sequence attached to the Au surface.

**Table 2:** Change of *R*_CT_ depending on the type of buffer solution and the identity of the G4 DNA sequences

Buffer	*c-KIT*	*c-MYC*	Htelo	*BCL2*
K^+^ only → Li^+^ and K^+^	–	–	no change	–
+ Pb^2+^	+ +	no change	+ +	+

## Supplementary Information

Below is the link to the electronic supplementary material.

Supplementary file1 (PDF 1174 KB)
