# Supplementary material for: Metal-dependent electrochemical discrimination of DNA quadruplex sequences
Source: J Biol Inorg Chem. 2021 Aug 4;26(6):659–66. doi: 10.1007/s00775-021-01881-9 (PMC8437839; doi:10.1007/s00775-021-01881-9)
Supplement: Supplementary file 1 — Supplementary file1 (PDF 1174 KB) [file 775_2021_1881_MOESM1_ESM.pdf]

Supplementary Material

Metal-dependent electrochemical discrimination of DNA quadruplex sequences

Daniela Escher,[a] M. Nur Hossain,[b] Heinz-Bernhard Kraatz*[b] and Jens Müller*[a]

[a] *Westfälische Wilhelms-Universität Münster, Institut für Anorganische und Analytische Chemie, Corrensstraße 28/30, 48149 Münster, Germany.*

[b] *University of Toronto Scarborough, Department of Physical and Environmental Sciences, 1265 Military Trail, Toronto M1C 1A4, Canada*

*Corresponding authors:

Email: mueller.j@uni-muenster.de and bernie.kraatz@utoronto.ca

Table of contents

1. MALDI-TOF MS spectra and data **S3**

Figure S1-1. Mass spectrum for the *c-KIT* sequence S3

Figure S1-2. Mass spectrum for the *c-MYC* sequence S3

Figure S1-3. Mass spectra for the HTelo sequence S3

Figure S1-4. Mass spectra for the *BCL2* sequence S4

Table S1-1. Overview of the G4 DNA sequences S4

2. CD spectroscopic studies **S5**

Figure S2-1. CD spectra for the *c-KIT* sequence S5

Figure S2-2. CD spectra for the *c-MYC* sequence S5

Figure S2-3. CD spectra for the HTelo sequence S5

Figure S2-4. CD spectra for the *BCL2* sequence S6

3. Electrochemical impedance spectroscopy experiments **S7**

Table S3-1. Summarized data for the equivalent circuit elements considering standard deviations S7

Table S3-2. Summarized data for the equivalent circuit elements considering the error of the fit S7

Figure S3-1. Charge transfer resistances represented in a bar diagram considering standard deviation or the error of the fit S8

Scheme S3-1. Equivalent circuit used in this study S8

Scheme S3-2. Alternative equivalent circuits S8

Table S3-3. Exemplary *F* tests comparing the modified circuit with the Randles circuit S9

Table S3-4. Exemplary *F* tests comparing the modified circuit with the modified Randles circuit S10

Figure S3-2. EIS data exemplarily fitted to three different equivalent circuits S11

Table S3-5. Selected fit parameters for the evaluation of a suitable equivalent circuit S12

Figure S3-3. EIS data for the measurements in K^+ buffer S13

Figure S3-4. EIS data for the measurements in Li^+ buffer S14

Figure S3-5. EIS data for the measurements in Li^+ buffer in the presence of Pb^{2+} S15

Figure S3-6. EIS data for the MCH-covered Au electrode S16

4. References **S16**

1. MALDI-TOF MS spectra and data

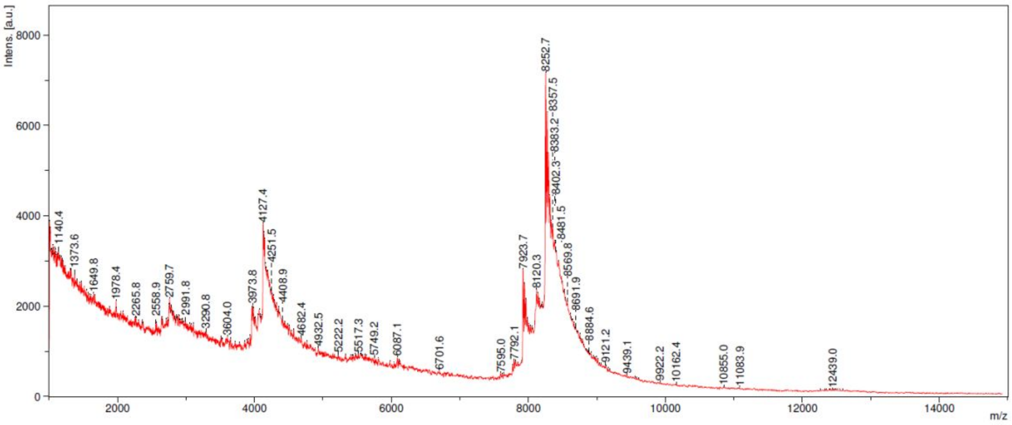

Figure S1-1. Mass spectrum of the *c-KIT* sequence. Calculated mass for [DNA+H]⁺: m/z = 8252.4 Da.

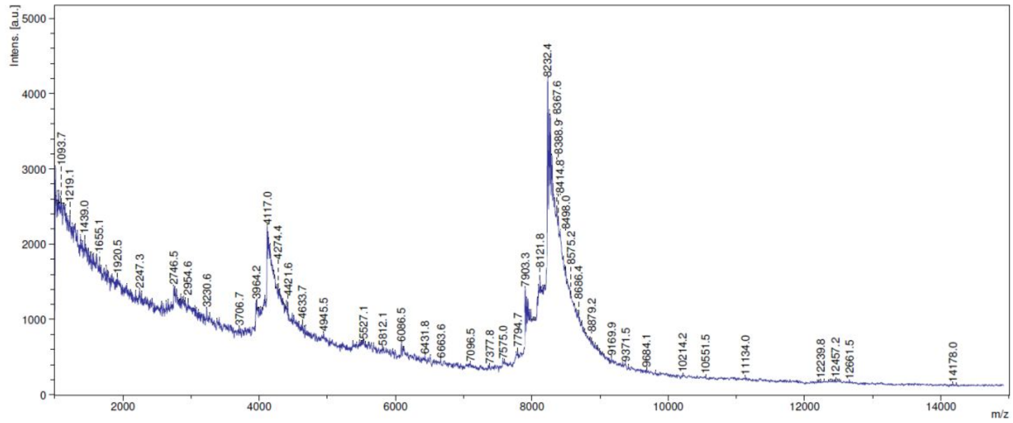

Figure S1-2. Mass spectrum of the *c-MYC* sequence. Calculated mass for [DNA+H]⁺: m/z = 8232.4 Da.

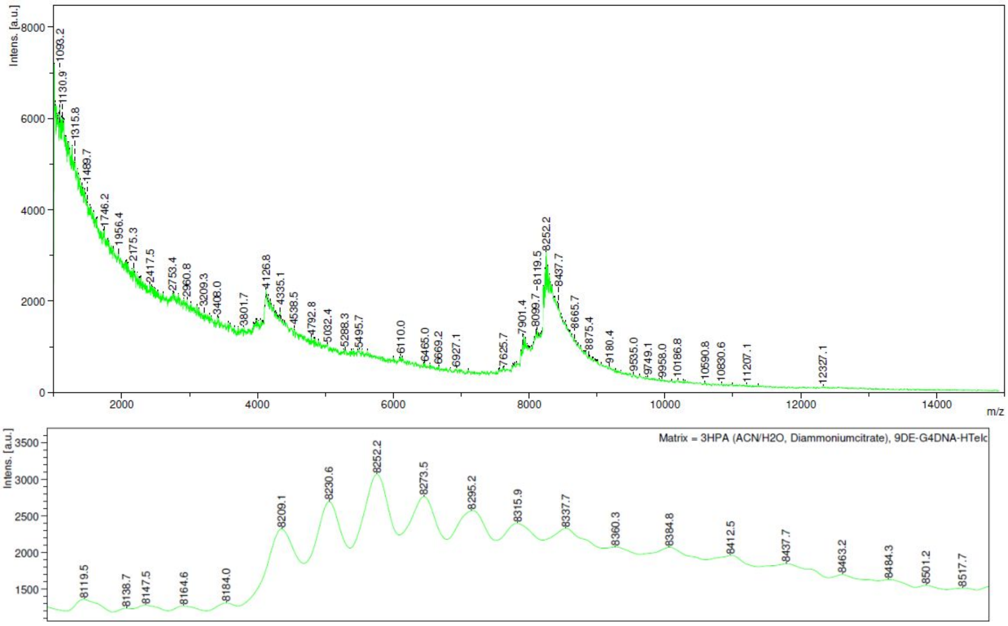

Figure S1-3. Mass spectrum (and expanded view) of the HTelo sequence. Calculated mass for [DNA+H]⁺: m/z = 8209.4 Da.

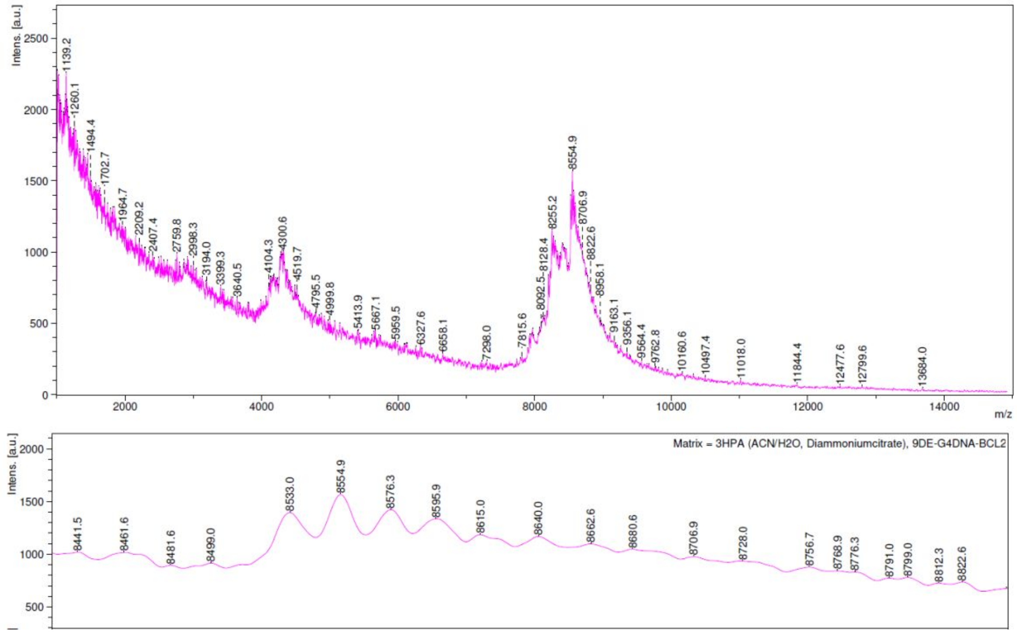

Figure S1-4. Mass spectra of the *BCL2* sequence. Calculated mass for $[DNA+H]^+$: m/z = 8532.5 Da.

Table S1-1. Overview of the G4 DNA sequences, their chemical formula and the calculated and experimental masses.

G4 DNA	Sequence	Chemical Formula	***m/z* / Da**	
			calcd. for $[DNA+H]^+$	found
c-KIT	R-5'-d(TTT AGG GAG GGC GCT GGG AGG AGG G)	$C_{260}H_{330}N_{109}O_{152}P_{25}S_2$	8252.4	8252.7
c-MYC	R-5'-d(TTT TGA GGG TGG GTA GGG TGG GTA A)	$C_{262}H_{334}N_{101}O_{156}P_{25}S_2$	8232.4	8232.4
HTelo	R-5'-d(TTT AGG GTT AGG GTT AGG GTT AGG G)	$C_{262}H_{335}N_{98}O_{157}P_{25}S_2$	8209.4	8209.1
BCL2	R-5'-d(TTT GGG CGC GGG AGG AAT TGG GCG GG)	$C_{269}H_{343}N_{109}O_{160}P_{26}S_2$	8532.5	8533.0

2. CD-spectroscopic studies

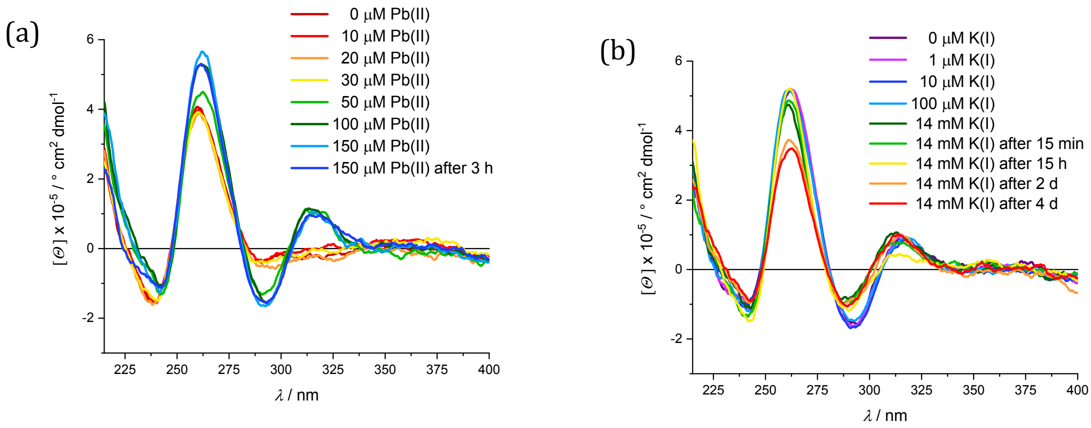

Figure S2-1. CD spectra of the Pb^{2+} titration for *c-KIT* in the presence of 60 mM Li^+ (a) and subsequent titration of K^+ to this solution (b). 14 mM K^+ equals the K^+ concentration of the 2 mM $K_4[Fe(CN)_6]/K_3[Fe(CN)_6]$ measurement solution of the EIS studies. 1 μM Pb^{2+} ≙ 1 equiv. with respect to the G4 DNA concentration of 1 μM.

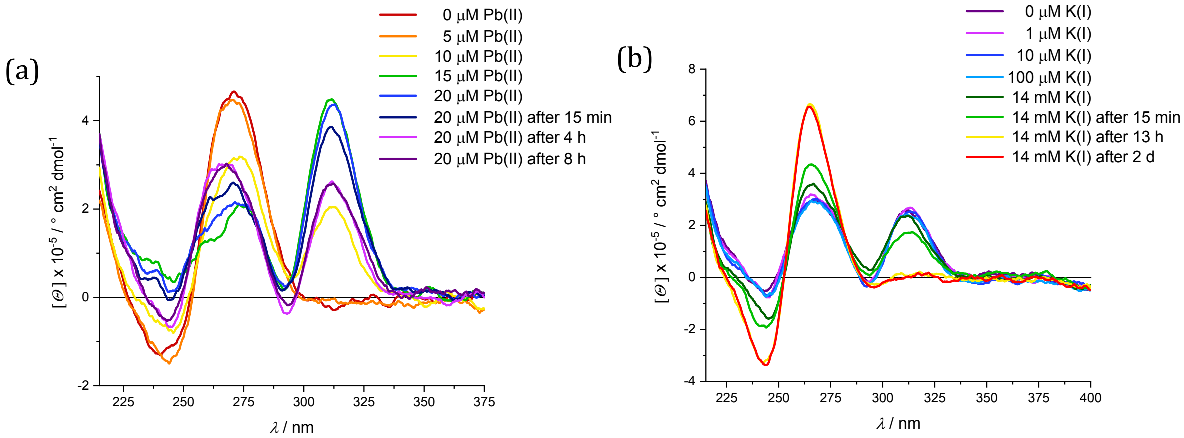

Figure S2-2. CD spectra of the Pb^{2+} titration for *c-MYC* in the presence of 60 mM Li^+ (a) and subsequent titration of K^+ to this solution (b). 14 mM K^+ equals the K^+ concentration of the 2 mM $K_4[Fe(CN)_6]/K_3[Fe(CN)_6]$ measurement solution of the EIS studies. 1 μM Pb^{2+} ≙ 1 equiv. with respect to the G4 DNA concentration of 1 μM.

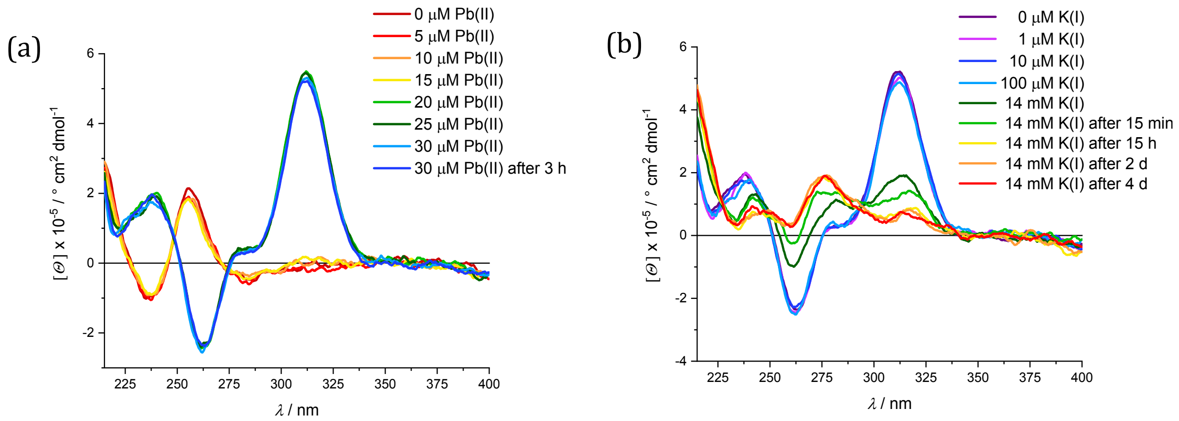

Figure S2-3. CD spectra of the Pb^{2+} titration for HTelo in the presence of 60 mM Li^+ (a) and subsequent titration of K^+ to this solution (b). 14 mM K^+ equals the K^+ concentration of the 2 mM $K_4[Fe(CN)_6]/K_3[Fe(CN)_6]$ measurement solution of the EIS studies. 1 μM Pb^{2+} ≙ 1 equiv. with respect to the G4 DNA concentration of 1 μM.

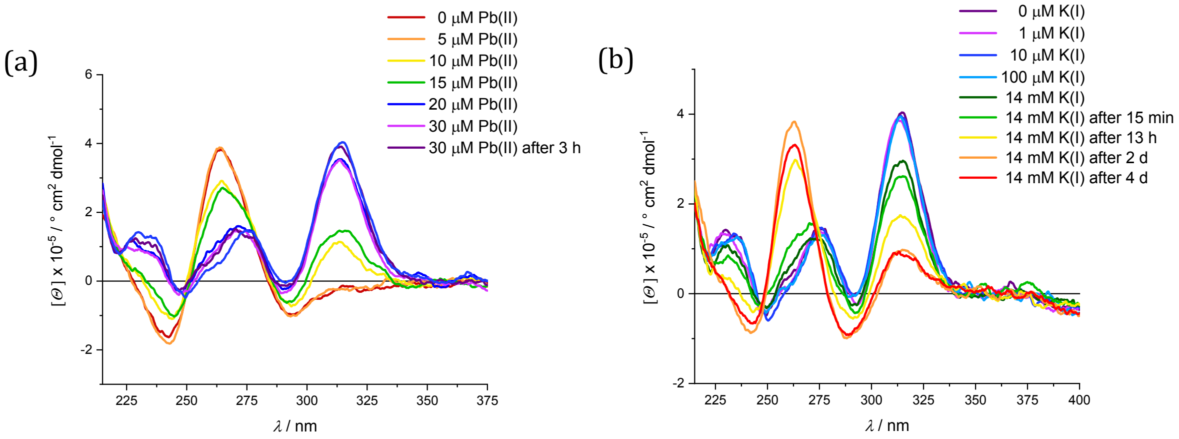

Figure S2-4. CD spectra of the Pb^{2+} titration for *BCL2* in the presence of 60 mM Li^{+} (a) and subsequent titration of K^{+} to this solution (b). 14 mM K^{+} equals the K^{+} concentration of the 2 mM $K_4[Fe(CN)_6]/K_3[Fe(CN)_6]$ measurement solution of the EIS studies. 1 μM Pb^{2+} ≙ 1 equiv. with respect to the G4 DNA concentration of 1 μM.

3. Electrochemical impedance spectroscopy experiments

Table S3-1. Values for the individual equivalent circuit elements for the different G4 DNA sequences in the presence of K^+, Li^+ (in the presence of 14 mM K^+) as well as Pb^{2+} (in the presence of 60 mM Li^+ and 14 mM K^+). Each value represents the average of three individual measurements with standard deviation. See Table S3-2 for an alternative treatment of the data to obtain a different error estimation and Figure S3-1 for a diagramed representation.

G4 DNA		C_{film} / nF	R_{CT} / kΩ cm²	R_x / kΩ cm²	CPE / μF	n_{CPE}
c-KIT	K^+	5.3±0.2	17±2	0.252±0.008	0.29±0.02	0.958±0.004
	Li^+	3.2±0.1	10.0±0.4	0.33±0.01	0.35±0.07	0.94±0.01
	Pb^{2+}	4.1±0.4	39±6	0.281±0.001	0.45±0.09	0.94±0.01
c-MYC	K^+	5.6±0.2	14±2	0.234±0.005	0.4±0.1	0.955±0.004
	Li^+	3.6±0.1	8.9±0.8	0.300±0.001	0.34±0.05	0.942±0.006
	Pb^{2+}	3.4±0.1	8.7±0.3	0.303±0.005	0.42±0.04	0.928±0.003
HTelo	K^+	5.0±0.1	23±2	0.248±0.001	0.30±0.04	0.952±0.007
	Li^+	3.4±0.1	28±4	0.313±0.002	0.47±0.03	0.94±0.01
	Pb^{2+}	4.1±0.5	53±4	0.28±0.01	0.33±0.04	0.96±0.01
BCL2	K^+	5.2±0.5	35±2	0.235±0.007	0.4±0.1	0.95±0.01
	Li^+	3.4±0.3	16±3	0.309±0.005	0.33±0.06	0.957±0.001
	Pb^{2+}	3.9±0.2	26±3	0.289±0.005	0.397±0.005	0.948±0.008

Table S3-2. Values for the individual equivalent circuit elements for the different G4 DNA sequences in the presence of K^+, Li^+ (in the presence of 14 mM K^+) as well as Pb^{2+} (in the presence of 60 mM Li^+ and 14 mM K^+). Each value represents the average of three individual measurements considering the error of the fit calculated by error propagation.

G4 DNA		C_{film} / nF	R_{CT} / kΩ cm²	R_x / kΩ cm²	CPE / μF	n_{CPE}
c-KIT	K^+	5.3±0.4	16.8±0.1	0.252±0.007	0.294±0.005	0.958± 0.002
	Li^+	3.2±0.3	10.0±0.1	0.33±0.01	0.349±0.006	0.939±0.002
	Pb^{2+}	4.1±0.3	38.7±0.2	0.28±0.01	0.451±0.005	0.936±0.002
c-MYC	K^+	5.6±0.4	13.8±0.1	0.234±0.007	0.427±0.007	0.955±0.002
	Li^+	3.6±0.3	8.90±0.05	0.30±0.01	0.345±0.006	0.942±0.002
	Pb^{2+}	3.4±0.3	8.74±0.05	0.30±0.01	0.424±0.007	0.928± 0.002
HTelo	K^+	5.0±0.4	23.2±0.1	0.248±0.007	0.302±0.005	0.952±0.002
	Li^+	3.4±0.3	27.7±0.1	0.31±0.01	0.466± 0.005	0.938±0.002
	Pb^{2+}	4.1± 0.4	53.1±0.3	0.28±0.01	0.333±0.005	0.961±0.002
BCL2	K^+	5.2±0.4	35.0±0.2	0.235±0.009	0.361±0.005	0.948±0.002
	Li^+	3.4±0.3	16.0±0.1	0.31±0.01	0.334±0.005	0.957±0.002
	Pb^{2+}	3.9±0.3	25.9±0.1	0.29±0.01	0.397±0.005	0.948±0.002

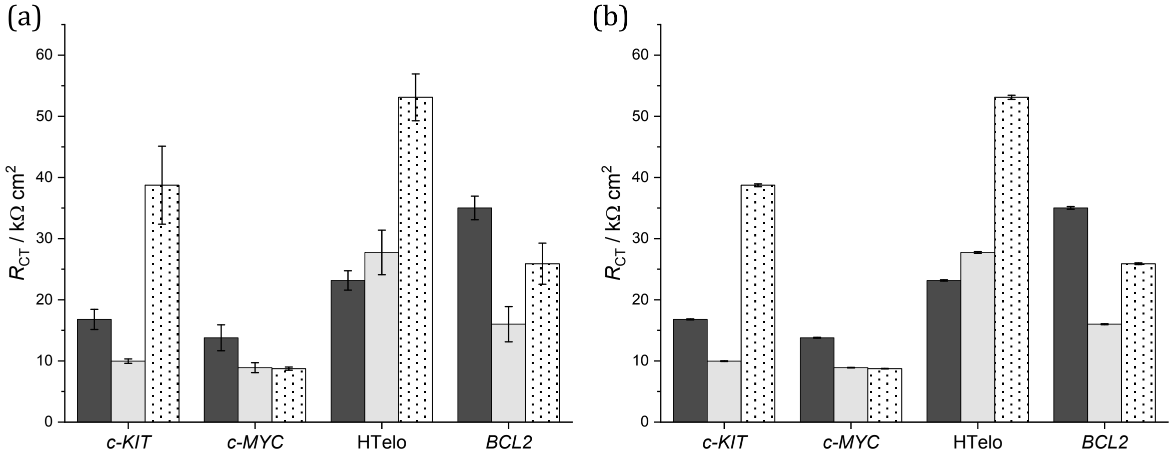

Figure S3-1. Charge transfer resistances of G4 DNA films on Au electrodes in the presence of K^+-containing buffer solution (dark gray), Li^+-containing buffer solution (light gray) or Li^+-containing buffer solution in the presence of Pb^{2+} (dotted). The data represent an average of three individual measurements considering standard deviations (a, Table S3-1) or the error of fitting the experimental data to the equivalent circuit shown in Scheme S3-1 (b, Table S3-2).

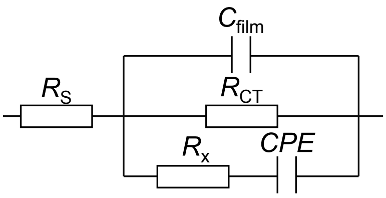

Scheme S3-1. Equivalent circuit used in this study. R_S represents the solution resistance, C_{film} the G4 DNA film capacitance and R_{CT} the charge transfer resistance. The additional resistor R_x and the constant phase element (*CPE*) take the inhomogeneity of the film on the Au electrode surface into account [1].

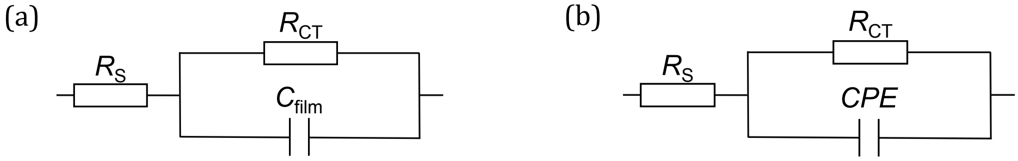

Scheme S3-2. Alternative equivalent circuits used for the fitting experiments (see Figure S3-1). a) $R_S(R_{CT}C_{film})$, b) $R_S(R_{CT}CPE)$.

Table S3-3a. Exemplary F test with the K^+ data set, comparing the modified circuit used in the manuscript with the Randles circuit.

DNA	S_1 for R(CR) (Scheme S3-2a)	S_2 for R(C(R(RQ))) (Scheme S3-1)	Number of frequencies (N)	F_{exp}	F_{theor} (95% confidence level)
c-KIT	446	0.59	54	25450	3
c-MYC	1153	2.15	59	20007	3
HTelo	418	0.52	57	28843	3
BCL2	397	0.67	57	21345	3

Table S3-3b. Exemplary F test with the Li^+ data set, comparing the modified circuit used in the manuscript with the Randles circuit.

DNA	S_1 for R(CR) (Scheme S3-2a)	S_2 for R(C(R(RQ))) (Scheme S3-1)	Number of frequencies (N)	F_{exp}	F_{theor} (95% confidence level)
c-KIT	664	0.145	48	136888	3
c-MYC	953	757	48	7.8	2.7
HTelo	1489	1285	58	5.8	2.7
BCL2	541	485	50	3.6	2.7

In both tables, S_1 and S_2 were calculated using equation 14.18 reported in: A. Lasia, *Electrochemical Impedance Spectroscopy and its Applications*, Springer New York, 2014.[2]

In both tables, F_{exp} was calculated following equation 14.25 reported in: A. Lasia, *Electrochemical Impedance Spectroscopy and its Applications*, Springer New York, 2014.[2]

k=3; m=3;

Degrees of freedom, model 1 (Scheme S3-2b): $2N - 3$

Degrees of freedom, model 2 (Scheme S3-1): $2N - 3 - 3$

$F_{exp} > F_{theor}$ with 95% confidence level

F_{exp} is larger than the theoretical value, so the addition of the three terms in model 2 are statistically justified at the confidence level assumed. Hence, the improvement is important and model 2 can be accepted.

Table S3-4a. Exemplary *F* test with the K^+ data set, comparing the modified circuit used in the manuscript with the modified Randles circuit.

DNA	S_1 for R(QR) (Scheme S3-2b)	S_2 for R(C(R(RQ))) (Scheme S3-1)	Number of frequencies (*N*)	F_{exp}	F_{theor} (95% confidence level)
c-KIT	196	0.59	54	16674	3
c-MYC	497	2.15	59	12894	3
HTelo	194	0.52	57	20072	3
BCL2	134	0.67	57	10800	3

Table S3-4b. Exemplary *F* test with the Li^+ data set, comparing the modified circuit used in the manuscript with the modified Randles circuit.

DNA	S_1 for R(QR) (Scheme S3-2b)	S_2 for R(C(R(RQ))) (Scheme S3-1)	Number of frequencies (*N*)	F_{exp}	F_{theor} (95% confidence level)
c-KIT	276	0.145	48	85382	3
c-MYC	334	757	48	-25	3
HTelo	478	1285	58	-35	3
BCL2	248	485	50	-23	3

S_1 and S_2 were calculated using equation 14.18 reported in: A. Lasia, *Electrochemical Impedance Spectroscopy and its Applications*, Springer New York, 2014.[2]

F_{exp} was calculated following equation 14.25 reported in: A. Lasia, *Electrochemical Impedance Spectroscopy and its Applications*, Springer New York, 2014.[2]

k=2; m=4;

Degrees of freedom, model 1 (Scheme S3-2b): $2N - 2$

Degrees of freedom, model 2 (Scheme S3-1): $2N - 4 - 2$

$F_{exp} > F_{theor}$ with 95% confidence level

F_{exp} is larger than the theoretical value, so the addition of the two terms in model 2 are statistically justified at the confidence level assumed for the measurements in K^+ buffer and for the measurements involving *c-KIT* in Li^+ buffer. Hence, the improvement is important and model 2 can be accepted in these cases. In the interpretation of the few remaining measurements, it was applied as well to allow a comparability of the parameters, despite the slight overfitting.

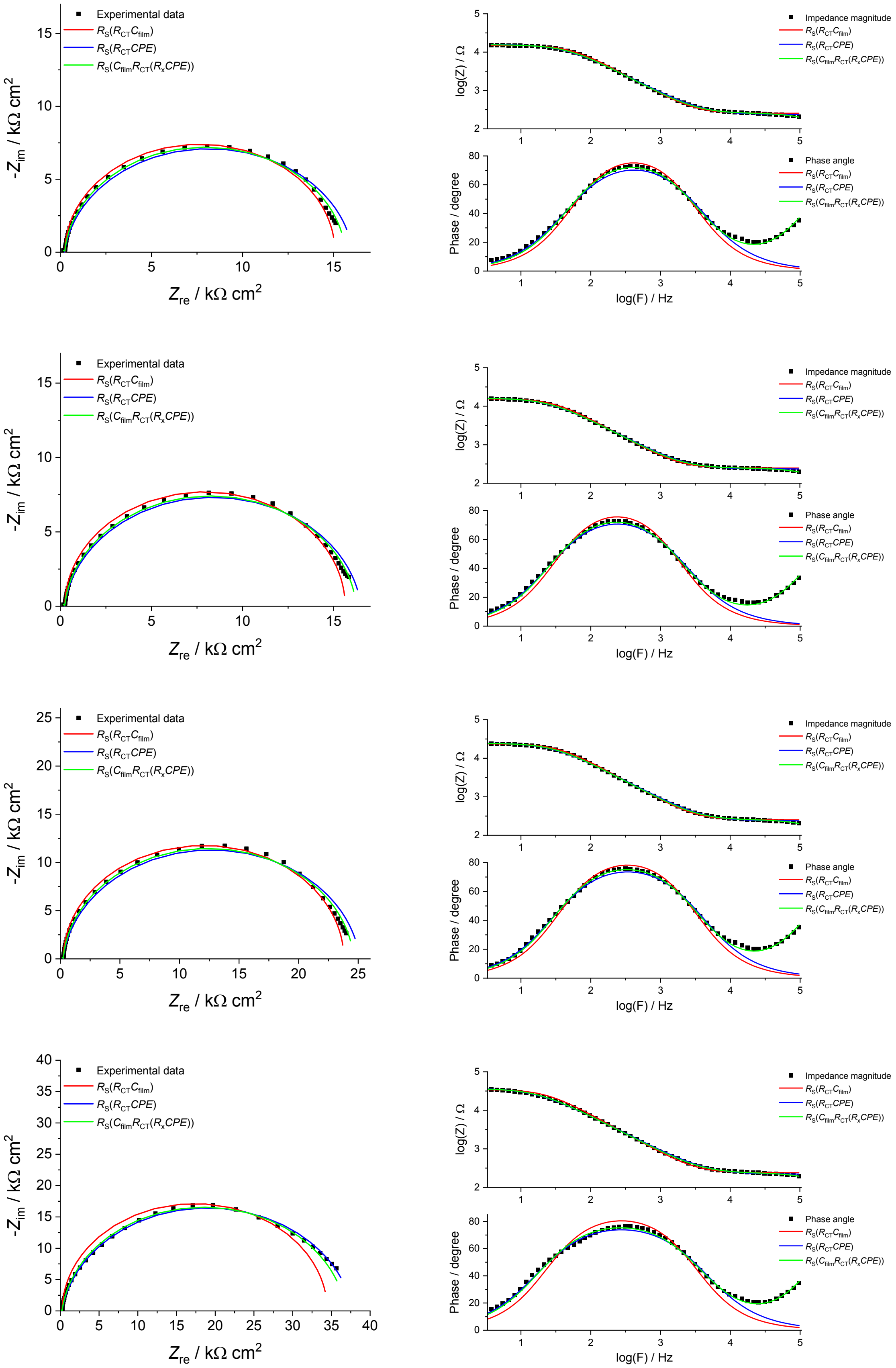

Figure S3-2. Nyquist plots (left) and Bode plots (right) for one example of *c-KIT* (a), *c-MYC* (b), HTelo (c) and *BCL2* (d) measured in K^+ buffer solution. The experimental data are fitted by three different equivalent circuits (red: $R_S(R_{CT}C_{film})$, blue: $R_S(R_{CT}CPE)$, green: $R_S(C_{film}R_{CT}(R_xCPE))$ shown in the Schemes S3-1 and S3-2.

Table S3-5. Selected fit parameters for the experimental EIS data of one measurement of *c-KIT*, *c-MYC*, HTelo and *BCL2* measured in K^+ buffer solution fitted to three different equivalent circuits ($R_S(R_{CT}C_{film})$, $R_S(R_{CT}CPE)$ and $R_S(C_{film}R_{CT}(R_xCPE))$, Schemes S3-1 and S3-2).

G4 DNA	Fit parameter	Equivalent circuit		
		$R_S(R_{CT}C_{film})$	$R_S(R_{CT}CPE)$	$R_S(C_{film}R_{CT}(R_xCPE))$
c-KIT	χ^2	$3.098\cdot10^{-2}$	$2.498\cdot10^{-2}$	$8.568\cdot10^{-4}$
	R_{CT} / kΩ cm^2	14.81	15.81	15.63
	% error	4.74	4.93	0.88
c-MYC	χ^2	$2.768\cdot10^{-2}$	$2.197\cdot10^{-2}$	$1.015\cdot10^{-3}$
	R_{CT} / kΩ cm^2	15.38	16.27	16.22
	% error	4.18	4.23	0.89
**HTelo**	χ^2	$2.920\cdot10^{-2}$	$2.340\cdot10^{-2}$	$8.749\cdot10^{-4}$
	R_{CT} / kΩ cm^2	23.54	24.89	24.60
	% error	4.46	4.51	0.85
BCL2	χ^2	$3.502\cdot10^{-2}$	$2.157\cdot10^{-2}$	$1.472\cdot10^{-3}$
	R_{CT} / kΩ cm^2	34.24	37.52	36.86
	% error	5.24	4.92	1.25

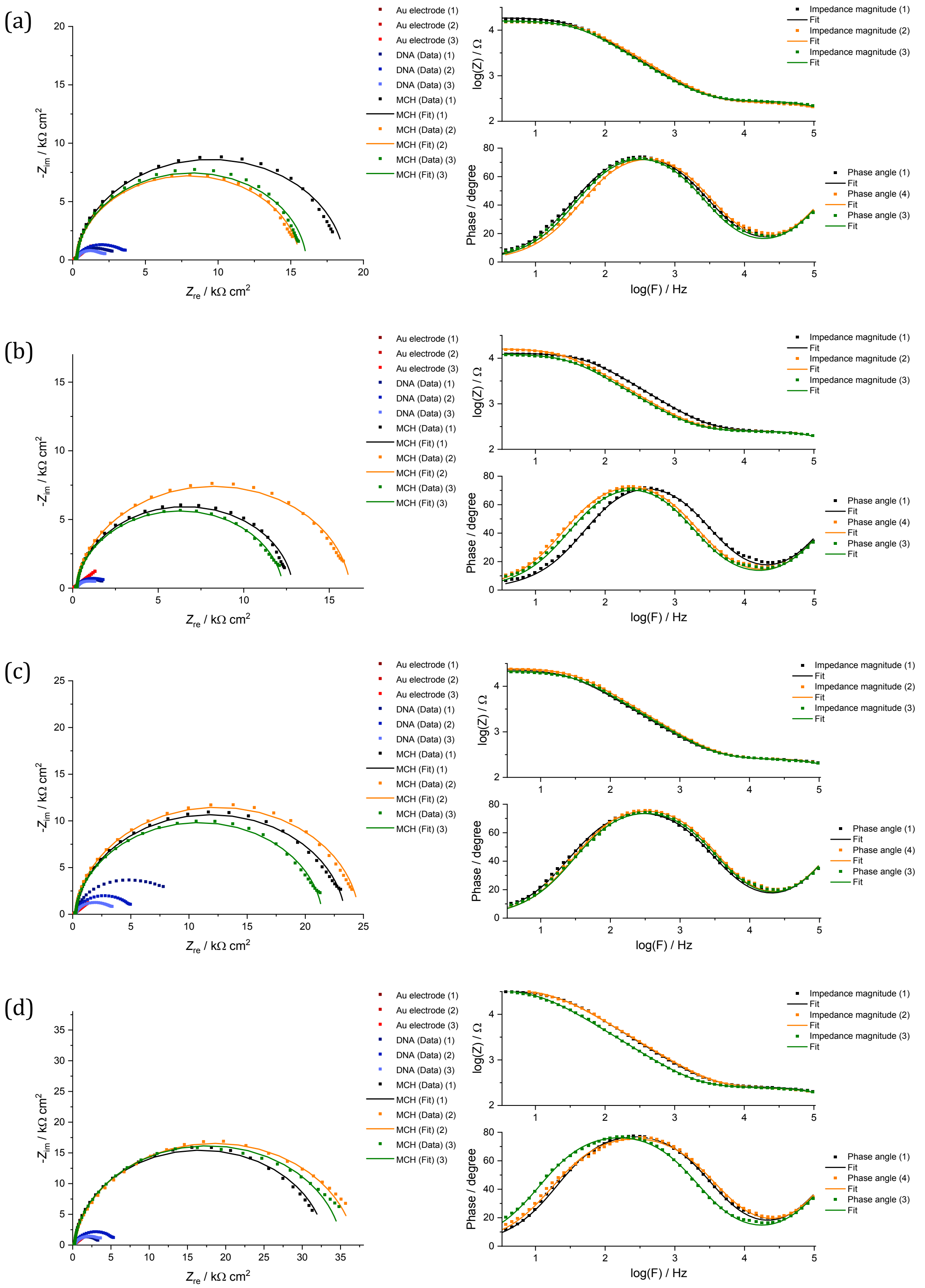

Figure S3-3. Nyquist plots (left) and Bode plots (right) of *c-KIT* (a), *c-MYC* (b), HTelo (c) and *BCL2* (d) measured in K^+ buffer solution. In the Nyquist plots, experimental data are shown for three individual measurements of the bare Au electrode (red), G4 DNA immobilized on the Au surface (blue) and after backfilling the DNA-covered electrodes with MCH (black/orange/green) with the corresponding fit to the equivalent circuit depicted in Scheme S3-1 (solid line). The Bode plots with fit are given for the MCH-filled G4 DNA film.

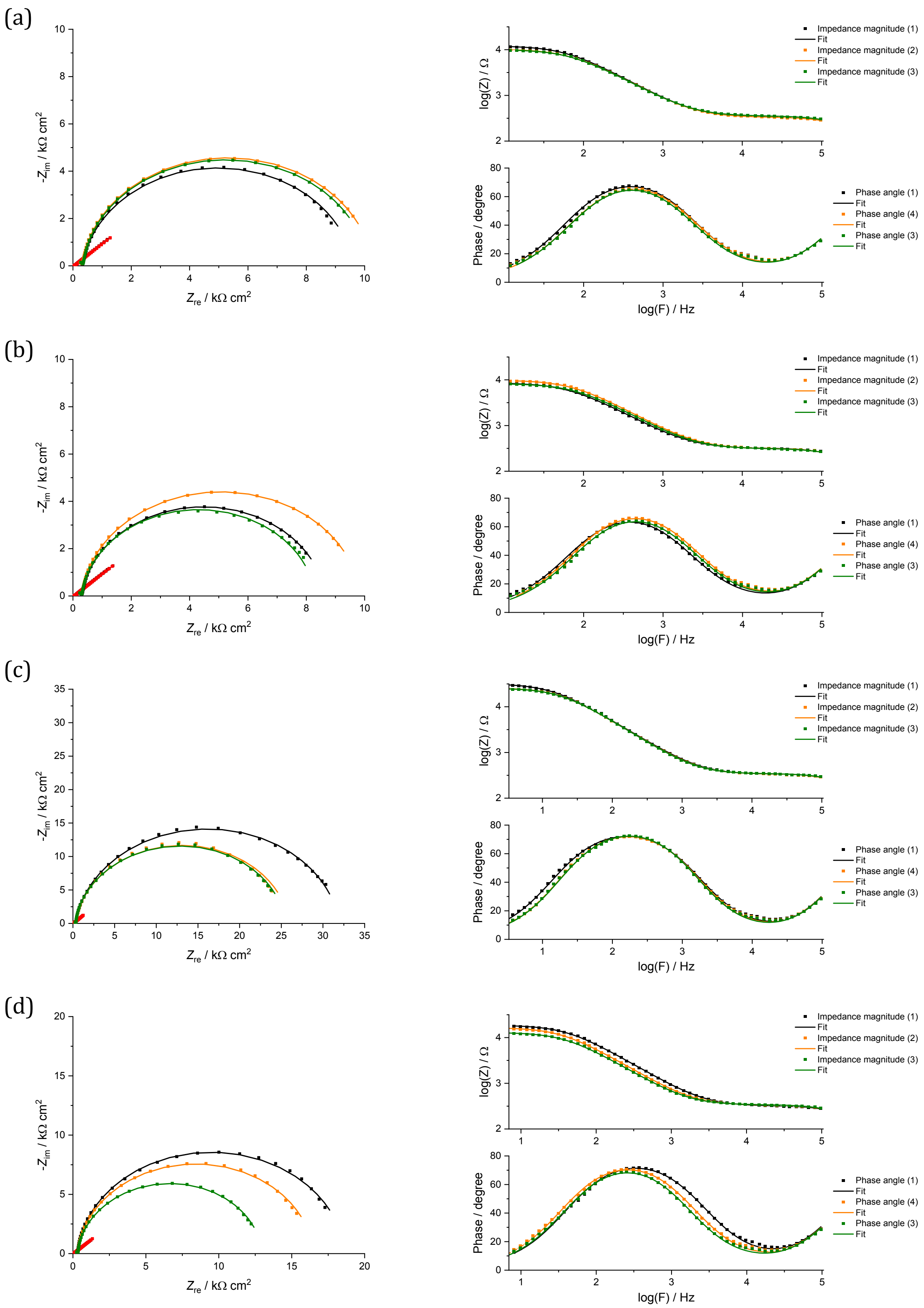

Figure S3-4. Nyquist plots (left) and Bode plots (right) of *c-KIT* (a), *c-MYC* (b), HTelo (c) and *BCL2* (d) measured in Li^+ buffer solution. In the Nyquist plots, experimental data are shown for three individual measurements of the bare Au electrode (red), G4 DNA immobilized on the Au surface (blue) and after backfilling the DNA-covered electrodes with MCH (black/orange/green) with the corresponding fit to the equivalent circuit depicted in Scheme S3-1 (solid line). The Bode plots with fit are given for the MCH-filled G4 DNA film.

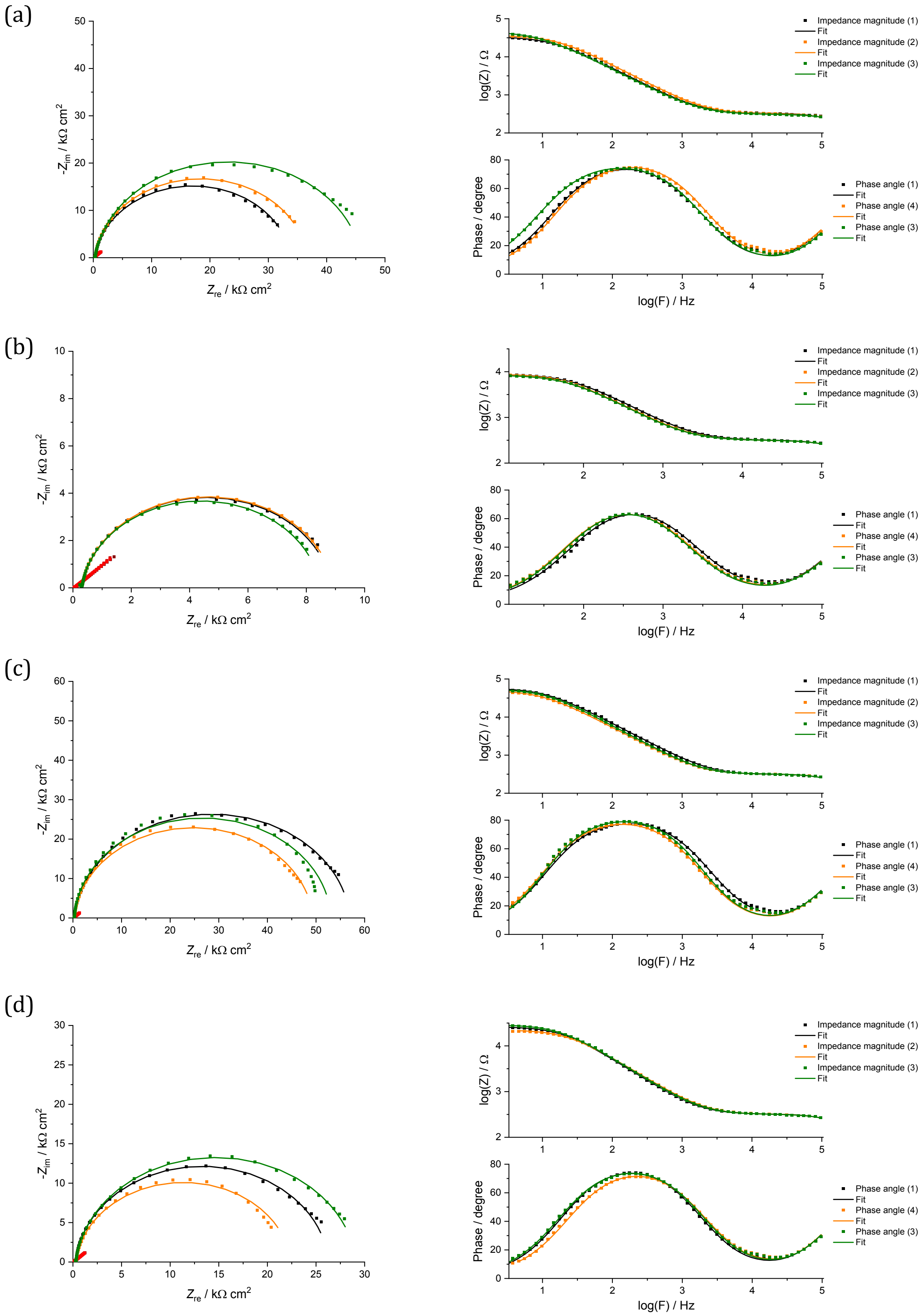

Figure S3-5. Nyquist plots (left) and Bode plots (right) of *c-KIT* (a), *c-MYC* (b), HTelo (c) and *BCL2* (d) measured in Li^{+} buffer solution after electrode incubation in the presence of Pb^{2+}. In the Nyquist plots, experimental data are shown for three individual measurements of the bare Au electrode (red), G4 DNA immobilized on the Au surface (blue) and after backfilling the DNA-covered electrodes with MCH (black/orange/green) with the corresponding fit to the equivalent circuit depicted in Scheme S3-1 (solid line). The Bode plots with fit are given for the MCH-filled G4 DNA film.

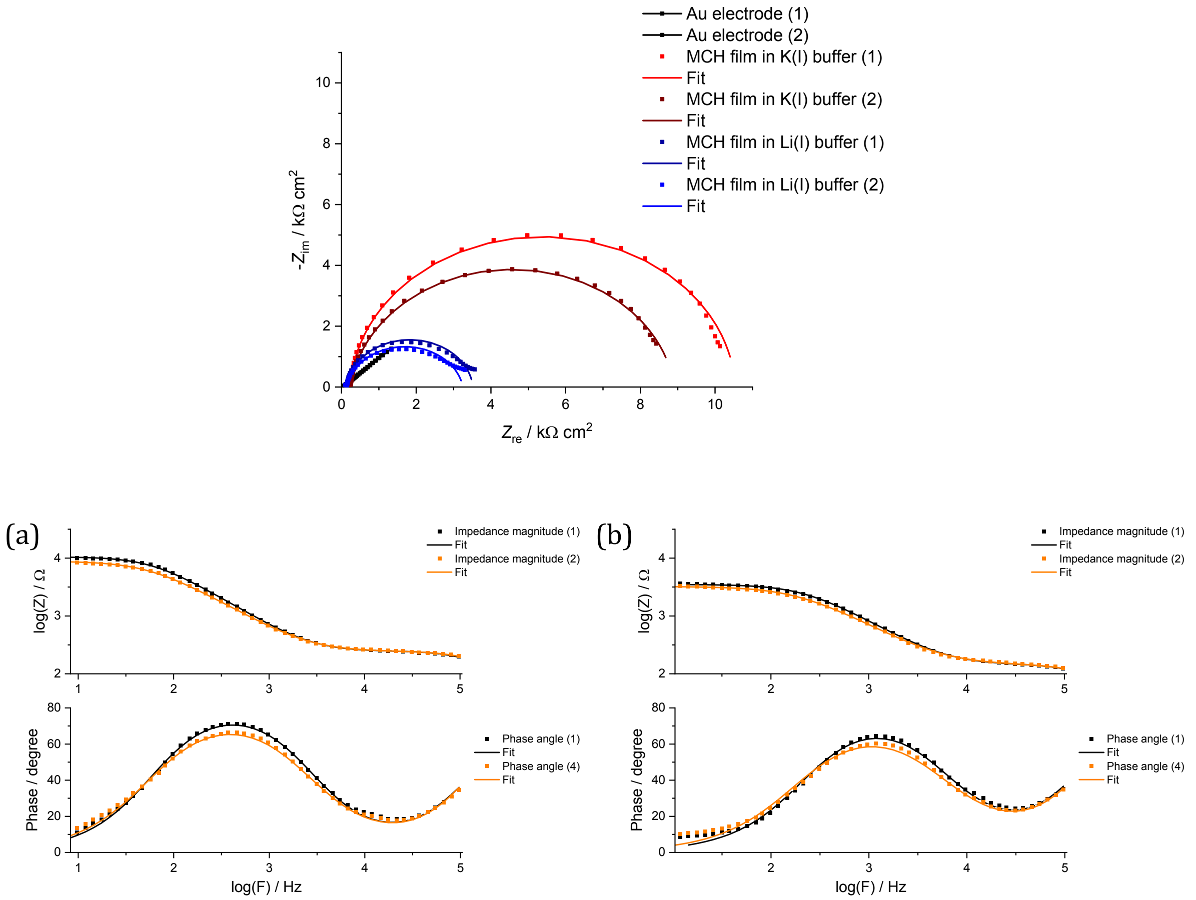

Figure S3-6. Nyquist plots (top) and Bode plots (bottom) of the MCH-covered Au electrode measured in K^+ buffer (a) or Li^+ buffer solution (b). The R_{CT} in K^+ buffer solution is 10.52±0.09 kΩ cm^2 (1) or 8.90±0.09 kΩ cm^2 (2), while the R_{CT} is smaller in Li^+ buffer solution with 3.52±0.04 kΩ cm^2 (1) or 3.25±0.05 kΩ cm^2 (2).

4. Reference

[1] Bin X, Kraatz H-B (2009) Interaction of metal ions and DNA films on gold surfaces: an electrochemical impedance study, Analyst 134:1309-1313.

[2] Lasia A, *Electrochemical Impedance Spectroscopy and its Applications*, Springer Science+Business Media, New York, **2014**.
